# Filamin A Binds to CCR2B and Regulates Its Internalization

**DOI:** 10.1371/journal.pone.0012212

**Published:** 2010-08-17

**Authors:** Laura Minsaas, Jesús Planagumà, Michael Madziva, Beate F. Krakstad, Míriam Masià-Balagué, Arieh A. Katz, Anna M. Aragay

**Affiliations:** 1 Department of Biomedicine, University of Bergen, Bergen, Norway; 2 Instituto de Biología Molecular de Barcelona, Consejo Superior de Investigaciones Científicas, Barcelona, Spain; 3 Division of Medical Biochemistry and Institute of Infectious Disease and Molecular Medicine, Faculty of Health Sciences, University of Cape Town, Cape Town, South Africa; University of Birmingham, United Kingdom

## Abstract

The chemokine (C-C motif) receptor 2B (CCR2B) is one of the two isoforms of the receptor for monocyte chemoattractant protein-1 (CCL2), the major chemoattractant for monocytes, involved in an array of chronic inflammatory diseases. Employing the yeast two-hybrid system, we identified the actin-binding protein filamin A (FLNa) as a protein that associates with the carboxyl-terminal tail of CCR2B. Co-immunoprecipitation experiments and *in vitro* pull down assays demonstrated that FLNa binds constitutively to CCR2B. The colocalization of endogenous CCR2B and filamin A was detected at the surface and in internalized vesicles of THP-1 cells. In addition, CCR2B and FLNa were colocalized in lamellipodia structures of CCR2B-expressing A7 cells. Expression of the receptor in filamin-deficient M2 cells together with siRNA experiments knocking down FLNa in HEK293 cells, demonstrated that lack of FLNa delays the internalization of the receptor. Furthermore, depletion of FLNa in THP-1 monocytes by RNA interference reduced the migration of cells in response to MCP-1. Therefore, FLNa emerges as an important protein for controlling the internalization and spatial localization of the CCR2B receptor in different dynamic membrane structures.

## Introduction

Chemokines and their receptors play an important role in the immune system by mediating translocation of leukocytes towards sites of inflammation [1]. The activation of chemokine receptors induces extensive cellular morphological changes through the rearrangement of the actin cytoskeleton, among other structures. Monocyte chemoattractant protein 1 (MCP-1/CCL2) is a chemokine secreted by numerous cell types including endothelial cells, epithelial cells, vascular smooth muscle and hematopoietic cells, and is a potent chemoattractant for monocytes and lymphocytes [1]. CCL2 is responsible for monocyte infiltration in a variety of chronic inflammatory diseases such as rheumatoid arthritis, atherosclerosis and multiple sclerosis, and has recently been implicated in cancer [2].

A key issue in the process of chemokine-induced cell migration is to understand the connection between chemokine receptor activation and cytoskeletal reorganization. Similar to other chemokine receptors, stimulation of the CCL2-receptor, CCR2, results in the activation of a plethora of intracellular signal transduction cascades, which leads to actin filament reorganization, cell polarization and cell movement [3]. The C-terminal intracellular domain of CCR2 is critical for mediating receptor desensitization and internalization [4]. Phosphorylation by GRK2 favors the recruitment of the arrestin proteins, leading to the subsequent uncoupling from G proteins and loss of receptor responsiveness [5], [6], [7]. Subsequently, the regulation by GRK and arrestin promotes clathrin-mediated internalization of inactivated receptors to endosomal compartments. Small changes in the levels of GRK2 expression can have a marked effect on the chemokine response [8], [9]. Interestingly, the protein FROUNT, which binds to the C-terminal tail of CCR2B, is involved in clustering of the receptor in the plasma membrane, which is important for chemotaxis [10]. Therefore, the C-terminus of CCR2B is indispensable for receptor endocytosis and recycling, and subsequent chemotaxis.

Filamin A (FLNa, cytoskeletal protein ABP-280) is an ubiquitously expressed dimeric actin cross-linking phosphoprotein which promotes orthogonal branching of actin filaments [11]. It also establishes critical links between the submembranous actin gel and integral membrane proteins, which stabilize the membrane, particularly during changes in cell shape associated with motility and migration. FLNa is composed of an N-terminal actin-binding domain, a C-terminal homodimerization domain, and a central rod-like backbone comprising 24 tandem repeats, each approximately 96 aa in length. Many different protein partners have been identified for FLNa. These include not only transmembrane proteins such as D2 and D3 dopamine receptors [12] and potassium channels [13], but also intracellular signaling molecules such as the Rho family of GTPases [14]. The ability to aggregate cytoskeletal elements, transmembrane receptors and cytoplasmic signaling proteins is potentially important not only in the stabilization of receptors at the cell surface, but also in cell signal integration and cell migration.

As mentioned above, the C-terminal tail of chemokine receptors is essential for receptor desensitization, internalization and chemotaxis. To search for novel proteins, which could interact with the CCR2B receptor, we employed the yeast two-hybrid assay using the C-terminal tail of the receptor as a bait to screen a human leukocyte cDNA library. We identified FLNa as an interacting partner of CCR2B and demonstrate that FLNa is essential for the initial step of receptor endocytosis after ligand stimulation. The identification of filamin A as a protein binding to the CCR2B sheds new light for understanding the physiological processes elicited by CCL2, and possibly, by other chemokines.

## Results

### The C-terminal tail of the CCR2B receptor interacts with the actin-binding protein, filamin A

**Figure 1:**
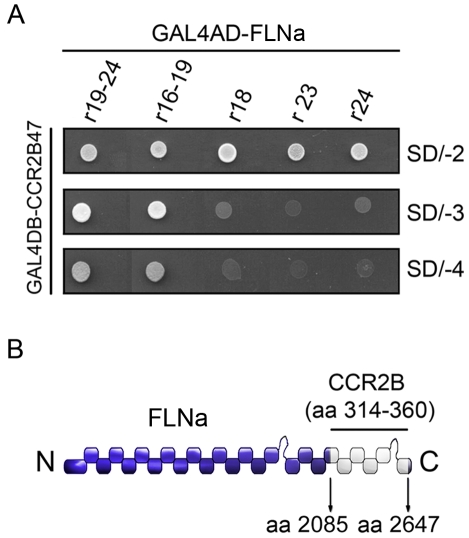
Interaction between CCR2B and FLNa in yeast two-hybrid. (**A**) The yeast expression plasmid pGBKT7-CCR2B47 (aa 314–360) was co-transformed into *S. cerevisiae* strain AH109 together with constructs encoding various repeats of human FLNa as indicated. (**B**) Schematic representation of human FLNa indicating that the epitope recognized by CCR2B maps between residues 2085–2647 of human FLNa.

A yeast two-hybrid approach was used to identify proteins that bind to the CCR2B receptor tail. Utilizing the pGBKT7-CCR2B47tri (aa 314–360) as bait, around 6×10^5^ independent colonies of a human leukocyte library were screened and multiple clones were isolated. To eliminate false positives, clones were re-streaked and several clones were rejected, since the interactions were found to be non-specific. One of the positive clones was found to encode a partial cDNA representing repeats 19–24 of FLNa (aa 2085–2647) (Figure 1A and B). FLNa is an actin-binding protein that regulates the assembly of actin filaments into dynamic 3D networks [15]. Interestingly, this region of FLNa, known as Rod 2 domain, has been found to bind various membrane proteins, among them several receptors [11]. To confirm the interaction between the FLNa peptide and the CCR2B-tail, pGBKT7-CCR2B47tri was co-transformed into *S. cerevisiae* strain AH109 together with the fragment found in the screening (repeats 19–24) and other fragments (16–19, 18, 23 or 24) of FLNa fused to the Gal4 activation domain. None of the filamin constructs or the receptor C-terminal tail supported growth by themselves. However, FLNa constructs encoding repeats 19–24 or 16–19, together with receptor C-terminal tail, supported growth, whereas individual repeats 18, 23 and 24 were negative (Figure 1A, middle and lowest panels) suggesting that the CCR2B-tail binds to a region of FLNa that comprises at least repeat 19.

**Figure 2:**
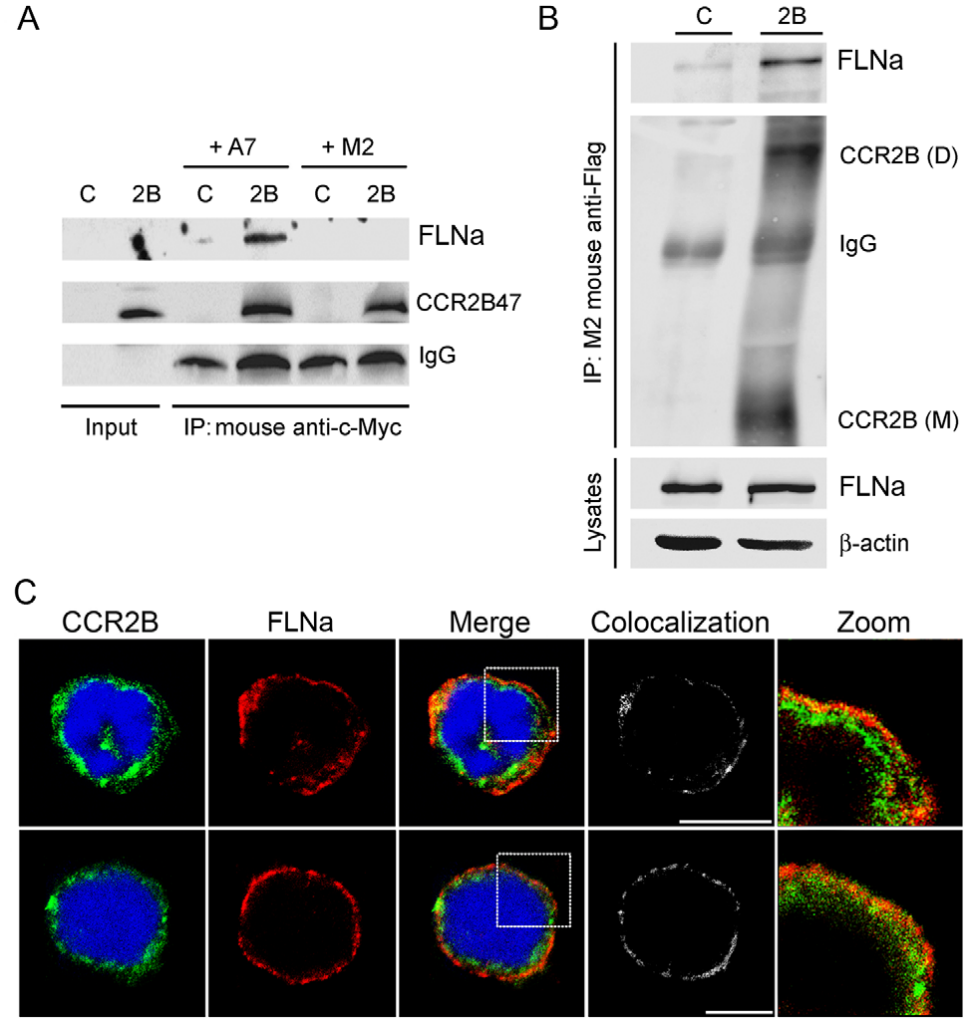
Interaction between CCR2B and FLNa and subcellular distribution of CCR2B with FLNa in monocytes. (**A**) Co-immunoprecipitation of the Myc-tagged CCR2B tail fragment and full length FLNa**.** Lysates from A7 and M2 cells were added to the *in vitro* transcription/translated CCR2B-tail (aa 314–360) and anti-c-Myc was used for immunoprecipitation. The translated parental expression vector was used as control (C). IgG levels are indicated in the lower panel. (**B**) Coimmunoprecipitation of full length CCR2B with endogenous FLNa. HEK293 cells were transfected with pcDNA3-FLAG-CCR2B (2B) or pcDNA3-FLAG (C). M2 anti-FLAG antibodies were used for immunoprecipitation. The CCR2B dimer (D) and monomer (M) are indicated. FLNa and β-actin levels in the lysates are shown in the two lower panels. Blots are representative of at least three independent experiments. (**C**) Mono Mac 1 cells (**upper panel**) and THP-1 (**lower panel**) cells were fixed and incubated with anti-CCR2 and donkey anti-rabbit Alexa Fluor 488 and anti-filamin 1 and Texas Red goat anti-mouse. Images are from one single layer of the Z stacks. The colocalization between CCR2B (green) and FLNa (red) was analyzed using Imaris colocalization software and is shown in white. Experiments were done in duplicates and repeated three times.

To determine whether the CCR2B-tail could interact with full-length FLNa, we analyzed the interaction between a CCR2B-tail peptide, synthesized in an *in vitro* transcription and translation system and FLNa protein present in A7 cells. M2-filamin A deficient cells were used as control. As can be seen (Figure 2A, upper panel), FLNa was pulled down with the CCR2B tail only in the presence of the A7 cell lysates, and no FLNa was immunoprecipitated from the M2 cell lysates. Further corroboration for the interaction was obtained by expressing the full length CCR2B tagged with the FLAG epitope in HEK293 cells and performing pull-down experiments to examine association with endogenous FLNa. Both monomer and dimer forms of the CCR2B were detected in the blot (Figure 2B, lane 2B). FLNa was co-immunoprecipitated together with CCR2B, whilst a very faint band was detected in cells transfected with empty vector (Figure 2B, lane C), probably due to unspecific antibody binding. Since the low levels of receptor expressed in monocytic cells precluded us from performing immunoprecipitation studies in those cells, we attempted to visualize the distribution of CCR2 and FLNa proteins in two human monocytic cell lines, Mono Mac 1 and THP-1, which endogenously express the CCR2 receptor [9] by immunofluorescence. Monocytes show expression of the receptor at the plasma membrane and also some diffuse staining in the cytoplasm (Figure 2C). FLNa colocalizes with the receptor preferentially at the plasma membrane in both cell lines. Taken together, these findings demonstrate that CCR2B associates with FLNa under basal conditions.

### Colocalization of CCR2B and FLNa in the leading edges of migrating cells

**Figure 3:**
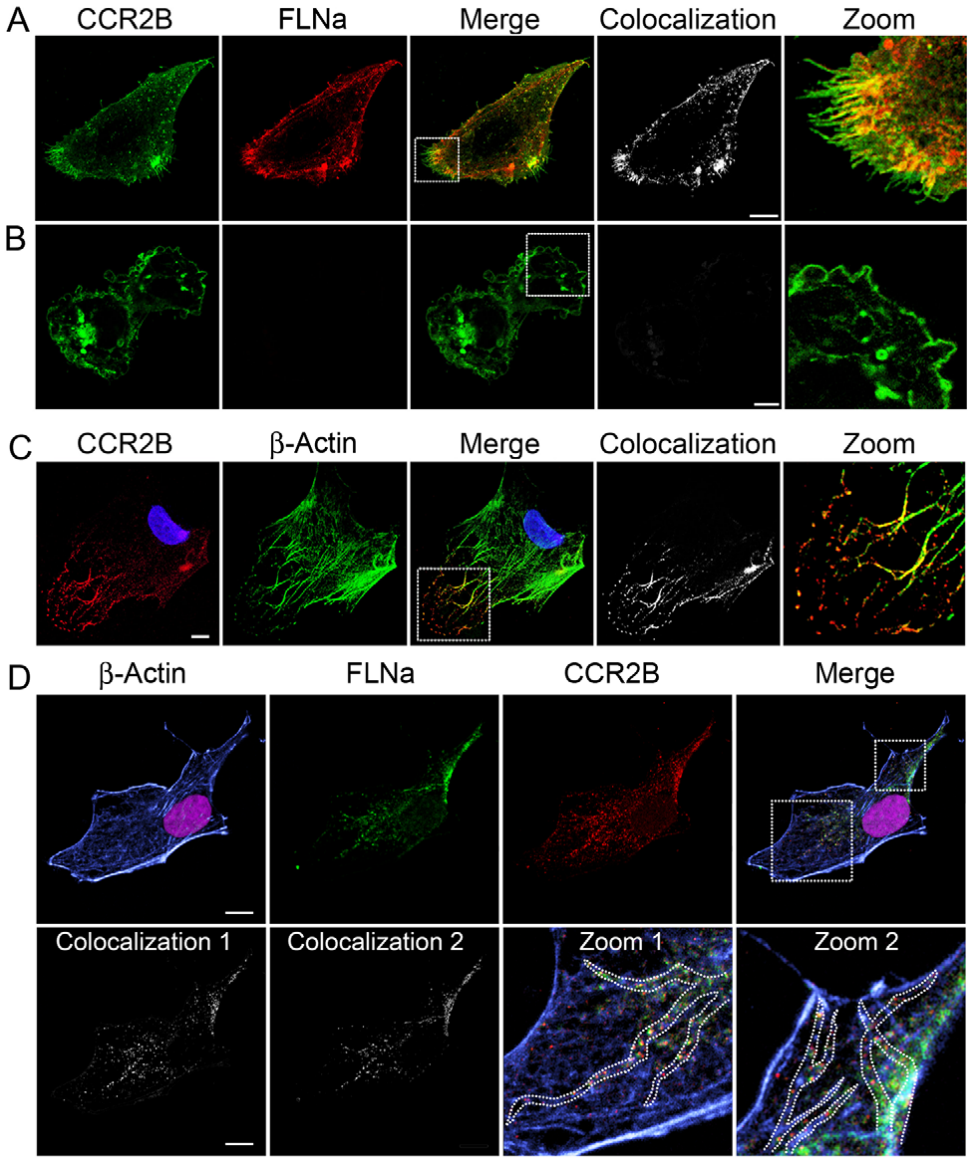
CCR2B localizes at the tips of actin filaments. CCR2B-A7 (**A**) and CCR2B-M2 (**B**) cells were fixed and incubated with antibodies and analyzed as described before. (**C**) CCR2B-A7 cells were fixed, permeabilized and stained with anti-FLAG Cy3 and FITC-phalloidin. Images are from one single layer of the Z stacks. The colocalization between CCR2B (red) and actin (green) was analyzed using Imaris colocalization software and is shown in white. Bars,10 µm. (**D**) CCR2B-A7 cells were fixed, permeabilized and stained with anti-FLAG Cy3, FITC-phalloidin, mouse anti-FLNa followed by goat anti-mouse Alexa633. Images show pseudo-colors for visualization purposes. Colocalization 1 (β-actin vs. CCR2B) and Colocalization 2 (FLNa vs. CCR2B) were analyzed using Imaris colocalization software and are shown in white. Dotted lines in zoom 1 and zoom 2 mark the direction of the stress fibers. Bars, 10 µm. Experiments were done in duplicates and repeated three times.

To investigate the possible function of FLNa, we studied the localization of FLAG-tagged CCR2B receptor in FLNa-repleted A7 cells and FLNA-deficient M2 cells. Figure 3A shows a representative A7 cell where the receptor is diffusely distributed on the cell surface. Some perinuclear and cytoplasmic staining of the receptor is also visible [10]. CCR2B and FLNa colocalized mainly at the plasma membrane and in filamentous structures found within the cells (see zoom image in Figure 3A). FLNa forms orthogonal intersecting actin filaments at the leading lamellae of cells [16]. Interestingly, the colocalization of the receptor and FLNa was prominent in lamellae at the leading edges of the cell. Accordingly, staining of cells for actin with phalloidin showed that CCR2B is associated with actin filament structures (Figure 3C) and CCR2B co-localizes with actin and FLNa at the leading edge of the cell, along the actin fibers and just at the tip of actin filaments (see Figure 3D colocalization 1 with actin and 2 with FLNa). Disruption of actin fibers with cytochalasin D (data not shown) did not disrupt the binding of FLNa to the receptor, but both proteins were relocated appearing in aggregates in the cell. These results suggest that filamin A links the receptor to actin fibers. As expected, M2 cells were negative for FLNa expression (Figure 3B). Surprisingly though, CCR2B was also present at the blebbing structures of the plasma membrane characteristic of the M2 cells (see detailed image in Figure 3B).

### Similar distribution of receptor in different sized clusters at the membrane

**Figure 4:**
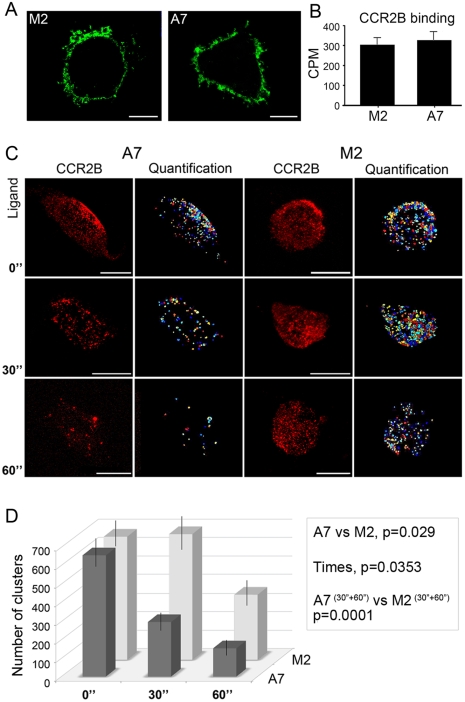
Equal levels of CCR2B in A7 and M2 cell surface by cluster analysis. (**A**) CCR2B-M2 and CCR2B-A7 cells incubated with anti-CCR2 and Alexa 488 anti-rabbit antibodies, were fixed without permeabilization. Bars, 10 µm. (**B**) Quantitative analysis of CCR2B binding at the surfaces of M2 and A7 cells by radioligand binding. The data represented by means ± SEM from three independent experiments, each performed in sextuplicates. (**C**) A7 and M2 cells transfected with pcDNA3-FLAG-CCR2B and stimulated with 20 nM CCL2 for the times indicated were incubated with anti-FLAG Cy3. Cells were fixed, permeabilized and treated with anti-rabbit Alexa Fluor 488. Z-stack Images were processed as maximum intensity projection and analyzed with CellProfiler software (**D**). Statistical analysis was done as described in the Methods. Clusters above 0.07 µm were quantified in M2 and A7 cells and plotted versus time after ligand addition. The graph represents the means ± SEM, n = 35 cells per cell type and p values of p<0.05. Bars,10 µm. Experiments were done in duplicates and repeated three times.

The fact that the receptor may also be present in the plasma membrane of filamin- deficient cells indicated that filamin A is not necessary for stabilizing the receptor at the membrane. In order to corroborate these results, M2 and A7 cells were immunostained with anti-CCR2 antibody (the epitome is present at the N-terminal tail of the receptor) without cell permeabilization, which allows the visualization of receptors present only at the plasma membrane. Figure 4A shows equal staining of receptors in both cell lines. Subsequently, whole cell binding with ^125^I-MCP-1 (CCL2) also demonstrated that the expression of the receptor in M2 and A7 cell membranes was equal (Figure 4B). Moreover, the affinity of the ligand for the CCR2 receptor was similar: Ki of 0.8±0.2 nM and 1.2±0.3 nM in A7 and M2 cells, respectively, which suggests that filamin A does not affect the affinity of the ligand for the receptor. These findings imply that filamin A is not necessary for membrane localization of the receptor and is not required for the activation of the receptor itself.

The chemokine receptors are distributed throughout the cell surface and after ligand stimulation, they are rapidly recruited into microdomain clusters at the plasma membrane [10]. Though FLNa does not seem necessary for plasma membrane localization it could help to induce clusters of receptor after ligand stimulation. Employing fluorescent microscopy to image the upper layers of non-permeabilized cells incubated with anti-FLAG antibody, we studied the clustering of CCR2B present at the membrane of A7 and M2 cells after CCL2 stimulation (Figure 4C) by quantifiying various size particles present in the membrane. Particles were divided in three different cluster sizes (R1 = 0.07−0.14 µm; R2 = 0.14−0.5 µm; R3 = 0.5−3 µm) (Figure 4C and Figure S1). Both cell lines showed a similar distribution of receptor in different sized clusters at the membrane, although a reduction in the overall number of particles upon ligand incubation was seen. Grouping all particles with sizes above 0.07 µm (Figure 4D) also indicated that the amount of surface-particles in A7 cells decreased rapidly with time in the presence of ligand. In contrast, the number of particles in M2 cells did not decrease so drastically. Overall, these results imply that FLNa is neither necessary for the localization of the receptor in the plasma membrane nor for receptor clustering after CCL2 stimulation, but they suggest that FLNa plays a specific role in CCL2-mediated internalization of CCR2B into vesicles.

### Filamin A is required for efficient internalization of the CCR2B receptor

Since our previous results showed a reduction in the levels of receptor in the plasma membrane after ligand addition whether FLNa was present or not, we investigated the possible role of FLNa in receptor endocytosis. First, the distribution of FLNa and the receptor was analyzed after CCL2-stimulation in THP-1 and A7-CCR2B cells (Figure S2A and B). CCL2 induced the redistribution of the receptor from a disperse staining into a punctate pattern in both THP-1 monocytes and A7-CCR2B cells. Receptor-enriched type vesicles were clearly seen at 30 and 60 min after ligand challenge, colocalizing with the punctate pattern of FLNa. In the case of A7 cells, only the receptors localized originally at the plasma membrane were followed. Initially, CCR2B was localized in ruffled structures at the membrane (Figure 3A). The vesicles containing the receptor and FLNa seemed to be located along the actin filament structures. Therefore, these results suggest that the receptor internalizes in endocytic vesicles upon CCL2 stimulation together with FLNa and that vesicles may travel along actin structures (Figure 3C zoom image).

**Figure 5:**
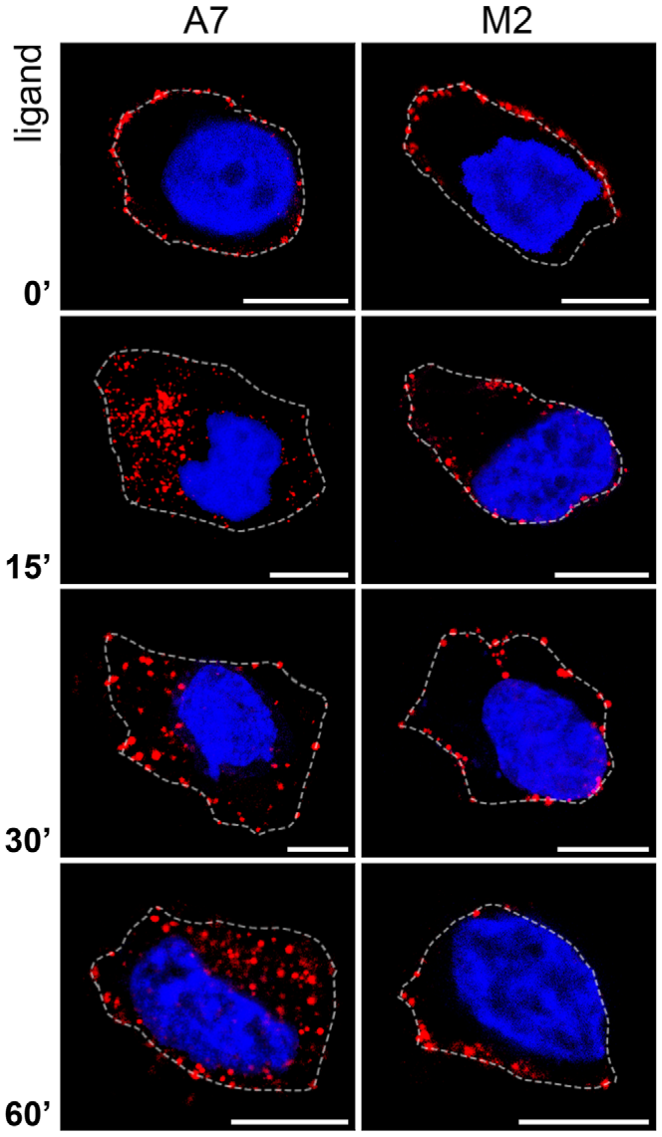
CCL2 activated CCR2B does not internalize efficiently in FLNa-depleted M2 cells. To follow the internalization of the CCR2B receptor, CCR2B-A7 and CCR2B-M2 cells grown onto coverslips were incubated on ice with anti-FLAG-Cy3 prior to incubation with 20 nM CCL2 for the times indicated. After this, cells were fixed and mounted. Images are representative of the majority of the cells and are from one single layer of Z stacks. White dotted lines show the boundaries of the cells. Experiments were done in duplicates and repeated three times. Bars,10 µm.

To study the requirement of filamin A in CCR2B endocytosis, the internalization of stimulated CCR2B in the FLNa-deficient M2 cells was followed and compared to the one in A7 cells. After 15 and 30 min of ligand incubation there was a poor internalization, if any, in M2 compared to A7 cells (Figure 5). A few cells showed some receptor in vesicles after ligand challenge but, on average, vesicles in the M2 cells were closer to the plasma membrane than in A7 cells and did not move deeper into the cell. Therefore, in the absence of FLNa there was little recruitment of the receptor to endocytic vesicles.

**Figure 6:**
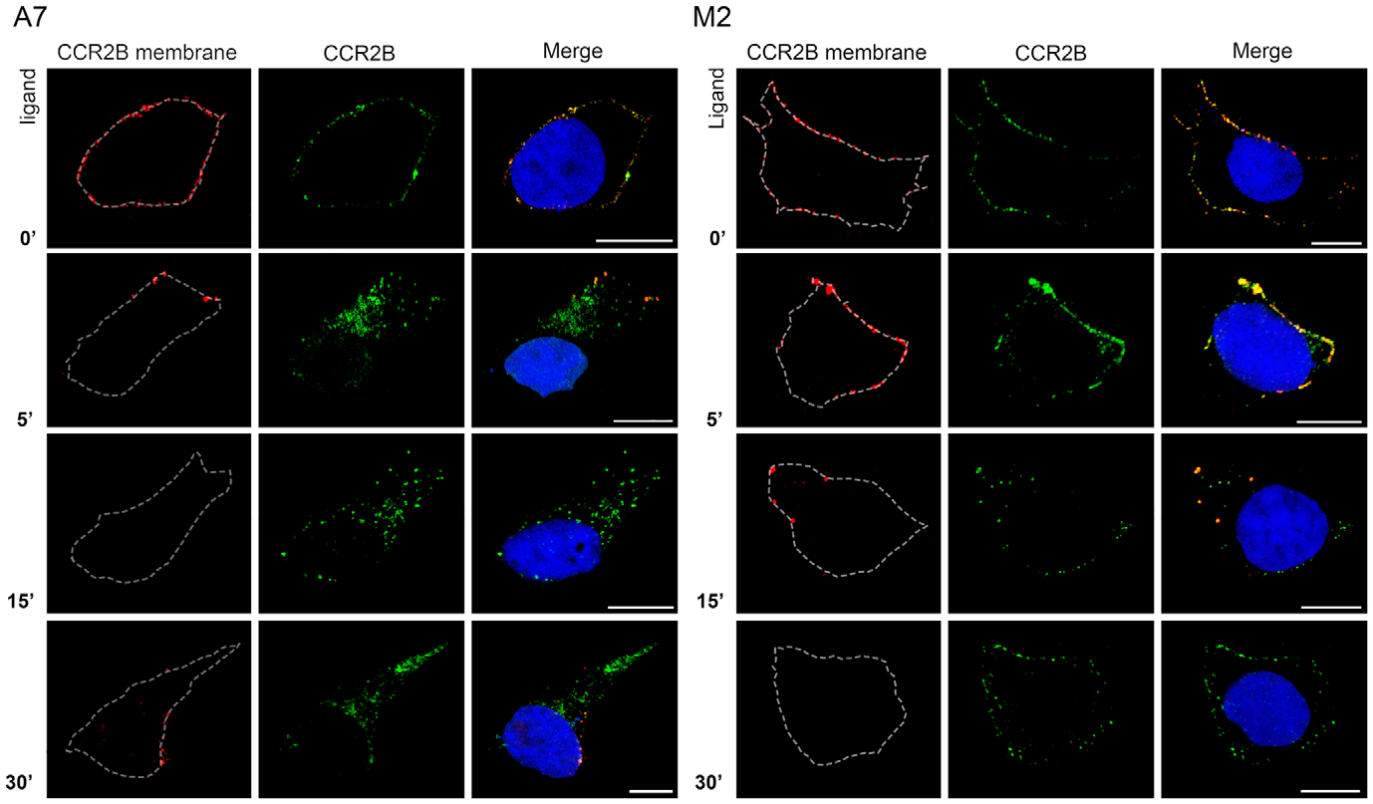
Internalization of the CCR2B receptor is delayed in absence of filamin. To visualize the receptors remaining at the plasma membrane, CCR2B-M2 and CCR2B-A7 were first incubated with 20 nM CCL2 for the times indicated and then treated with anti-FLAG-Cy3 prior to permeabilization. After this, cells were permeabilized and treated with anti-FLAG-FITC antibody. Non-internalized receptors present at the plasma membrane were detected in red and internalized receptor appears in green. Images are representative of the majority of the cells and are from one single layer of the Z stacks. White dotted lines show the boundaries of the cells. Experiments were done in duplicates and repeated three times. Bars,10 µm.

**Figure 7:**
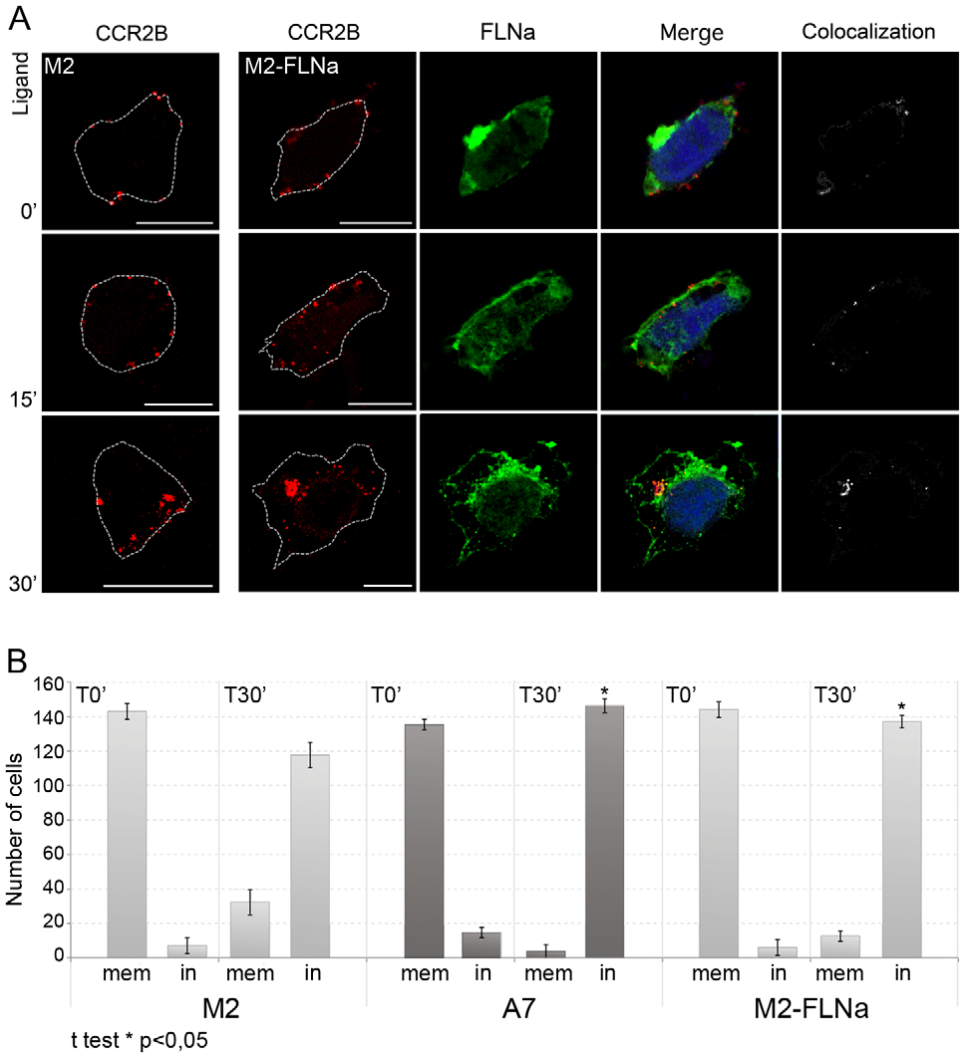
The internalization rate of the CCL2-stimulated CCR2B is increased in FLNa-repleted M2 cells. (**A**) M2 cells were co-transfected with pcDNA3-FLAG-CCR2B and pREP4-FLNa. Cells were further incubated with CCL2 for the times indicated and stained for CCR2B (red) and FLNa (green). Colocalization is shown in white. Left panel shows control CCR2B-M2 cells without FLNa. Images are from one single layer of the Z stacks. Experiments were done in duplicates and repeated three times. Bars, 10 µm. White dotted lines show the contour of the cells. (**B**) Quantification of exogenous CCR2B in the cell membrane (mem) or inside (in) M2, A7 and M2-FLNa cells plotted as mean results ± SEM (n = 450 per time point).*: p<0.05 for M2 T30′ (in) vs. A7 T30′(in) or M2-FLNa T30′ (in).

This effect could be due to a slower and inefficient internalization of the receptor from the plasma membrane to endocytic vesicles, which would leave some receptors at the membrane, and/or to an impairment of vesicle transport after the initial internalization from the membrane. To discern between the two processes, internalization or vesicle transport, we followed the receptors remaining at the plasma membrane after ligand stimulation. We labeled the receptors remaining at the plasma membrane after ligand stimulation with anti-FLAG-Cy3 antibodies prior to cell permeabilization, whilst total receptor in the cell was detected utilizing anti-FLAG-FITC antibodies after ligand addition and cell permeabilization. The receptors that did not undergo internalization after ligand stimulation would appear as red or yellow. As expected (Figure 6), unstimulated A7 and M2 cells had receptors stained with both antibodies at the plasma membrane (yellow staining). Fifteen minutes after addition of ligand, all the receptors were internalized in A7 cells. Interestingly, the red label reappeared in A7 cells at 30 min, which indicates that receptors were present again at the plasma membrane, probably due to receptor recycling. In contrast, M2 cells showed a marked delay in the internalization since the receptor was present at the plasma membrane even after 15 min of stimulation (see yellow dots at the periphery of the cell). Some receptor did eventually internalize and the dotted pattern was seen close to the plasma membrane. In order to confirm these results, FLNa deficient M2 cells were restored for FLNa by transient transfection (Figure 7). M2 cells that expressed FLNa (M2-FLNa) had more internalized particles than M2 cells at 30 min after ligand challenge. In fact, quantification of particles that were either at the plasma membrane or inside the cell (i.e. close to or not close to the membrane) showed that 30 min after stimulation there were significantly more particles remaining at the plasma membrane in M2 cells compared to A7 and M2-FLNa cells (Figure 7B). Also worth noticing, was that the internalized particles in M2-FLNa cells again contained both receptor and FLNa. These results, together with previous data, demonstrate that FLNa is necessary for efficient and rapid internalization from the plasma membrane to endosomes.

**Figure 8:**
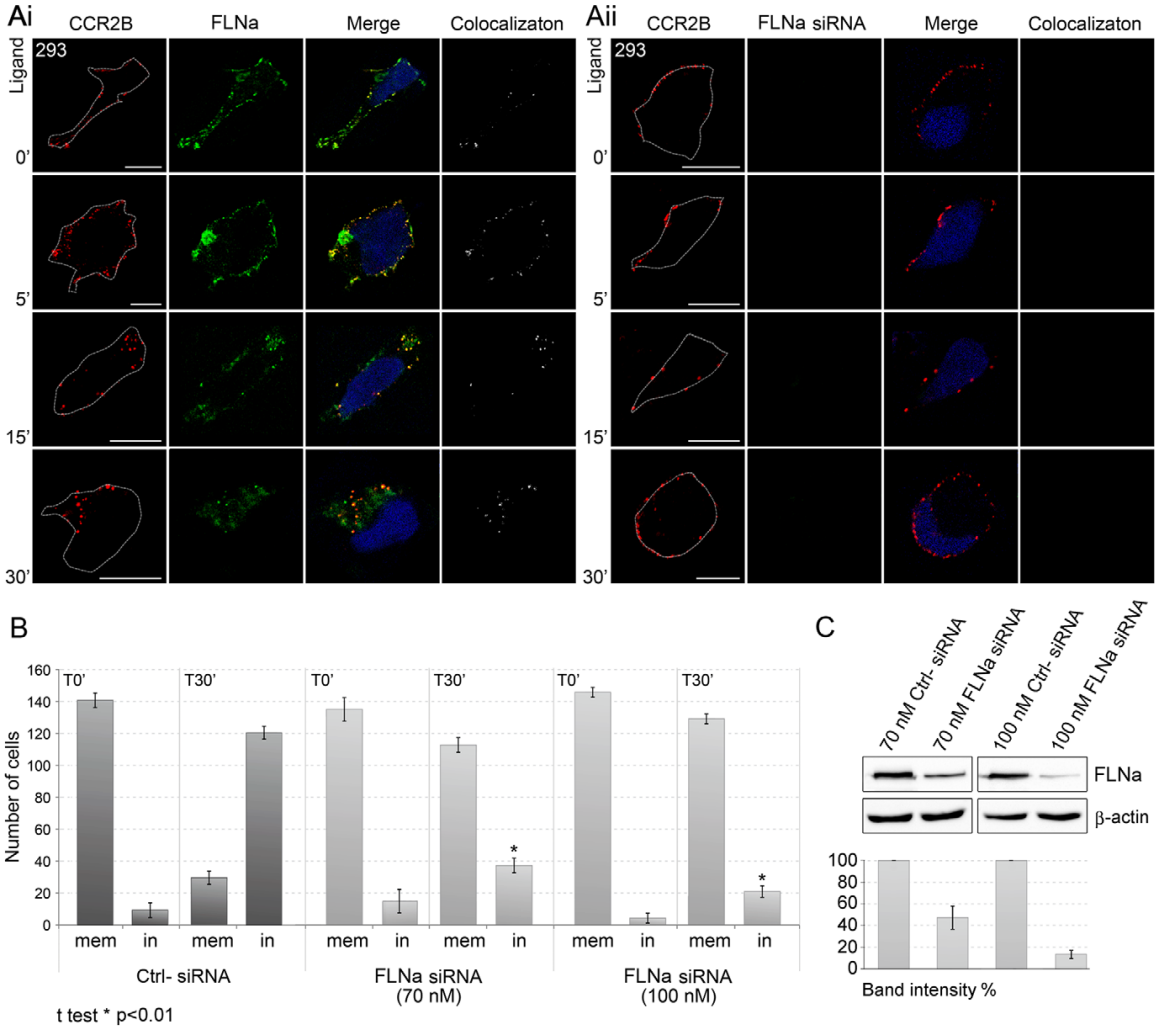
The internalization of the CCL2-stimulated CCR2B is significantly reduced after treatment with FLNa siRNA. (**A**) HEK293-CCR2B cells were treated with 100 nM of FLNa siRNA (**Ai**) or negative control siRNA (**Aii**). Cells were further incubated with CCL2 for the times indicated and stained for CCR2B (red) and FLNa (green). Colocalization is shown in white. Images are from one single layer of the Z stacks. Experiments were done in duplicates and repeated three times. Bars,10 µm. White dotted lines indicate the contour of the cells. (**B**) Quantification of CCR2B in the cell membrane (mem) or inside (in) the cell and plotted as mean results ± SEM (n = 450 per time point). *: p<0,01 for Ctrl- siRNA (100nM) T30′ (in) vs. FLNa siRNA (70 nM) T30′ (in) or FLNa siRNA (100 nM) T30′ (in). Data are from cells treated with 70 nM or 100 nM siRNA. (**C**) Immunoblot quantification of FLNa knock-down in HEK293-CCR2B cells treated with 70 nM or 100 nM siRNA as indicated. Histogram represents the mean intensity of the FLNa bands, normalized against the corresponding β-actin bands ± SEM. Experiments were repeated twice with triplicate samples.

Further evidence for the need of FLNa in receptor internalization came from experiments where FLNa was knocked-down by RNAi-mediated suppression using short interfering RNA (siRNA). HEK293-CCR2B cells showed a reduction in the level of the protein both in western blots and under confocal microscopy only when transfected with FLNa siRNA but not with two different control siRNAs (Figure 8 Aii and C). The depletion of FLNa by siRNA blocked the internalization of CCR2B even at 30 min after ligand stimulation (compare Figure 8 Ai to Aii). Quantification and sorting of cells either as having receptor mainly in the membrane or mainly in internalized particles, indicated that silencing of FLNa expression completely altered the share of cells with receptor in the membrane, an effect that was not observed in presence of control siRNA (Figure 8 Ai, Aii and B). In HEK293-CCR2B cells, FLNa once again colocalized together with the receptor in internalized vesicles. Since cells treated with siRNA against FLNa left the CCR2B receptor mainly at the plasma membrane after ligand stimulation, we analyzed the activation of intracellular calcium release, a well-known downstream cascade of CCR2. Both cells treated with siRNA against FLNa or control siRNA responded to CCL2 by elevating their intracellular calcium levels (Figure S3). The levels of calcium increase were similar in these cells supporting the idea that the receptor is activated in absence of FLNa and desensitized by GRK2. Taken together these results demonstrate that FLNa is necessary for the efficient internalization of ligand-stimulated receptor into endocytic vesicles.

### The binding of β-arrestin-2 to stimulated CCR2B is not affected by the lack of FLNa

Arrestin proteins promote clathrin-mediated internalization of inactivated receptors to endosomal compartments. We previously reported that agonist-induced endocytosis of CCR2B is GRK2 and β-arrestin-2-dependent [9]. Since the M2-FLNa depleted cells showed impairment in receptor internalization, we investigated the pattern of localization of receptor and β-arrestin-2 in both A7 and M2 cells. Internalization of the receptor was followed with FLAG-Cy3 pre-labeled antibody in cells expressing β-arrestin-2-GFP. After stimulation, the CCR2B receptor localized in endocytic vesicles as seen before. β-arrestin-2-GFP, which was diffusely present in the cytoplasm without stimulation, relocated to vesicles together with the receptor in both A7 and M2 cells (Figure S4). Though in M2 cells, vesicles were clearly located closer to the plasma membrane at late times. The binding of β-arrestin-2 to the stimulated receptor seems not to be affected by the lack of FLNa. Thus, the slow rate of receptor internalization cannot be due to impairment in β-arrestin-2 binding to the activated receptor.

### FLNa is necessary for CCL2-induced monocyte migration

**Figure 9:**
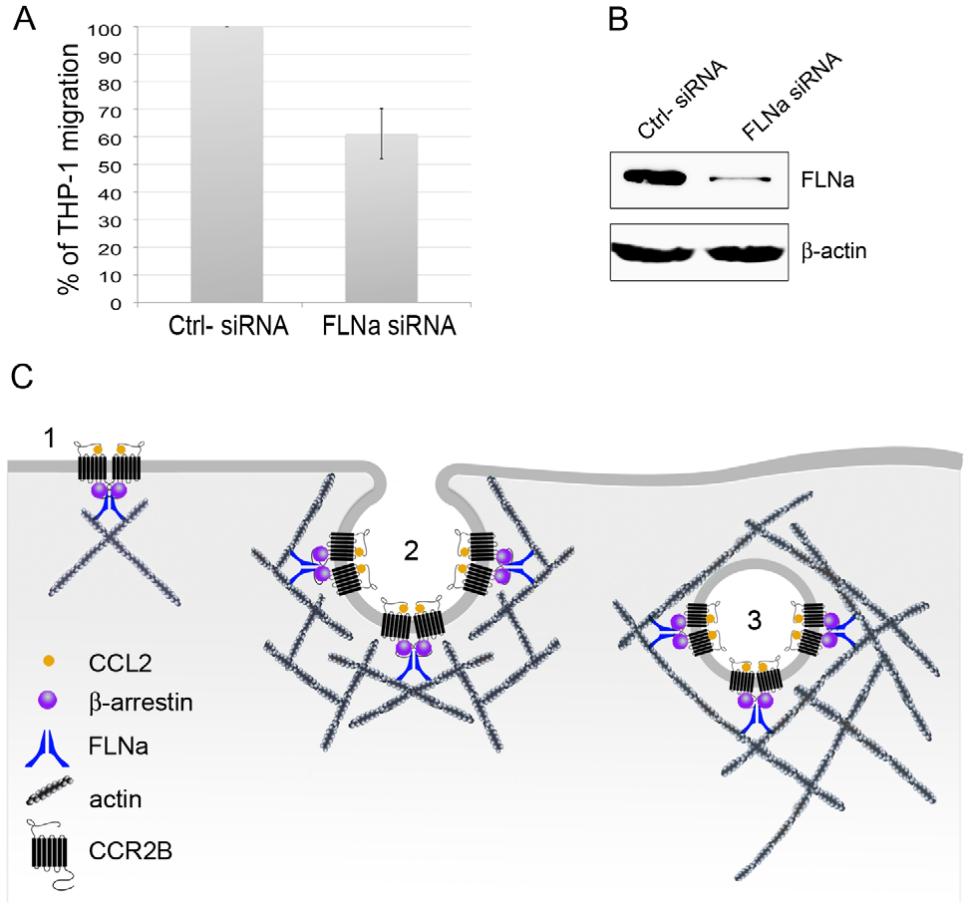
Chemotaxis in response to CCL2 is significantly reduced in THP-1 cells treated with FLNa siRNA. (**A**) Histogram of mean chemotaxis migration results from THP-1 cells treated for 5 days with 100 nM of FLNa siRNA or negative control siRNA. Cells were left to migrate for 24 hours. Experiments were repeated four times. (**B**) Immunoblot of FLNa knock-down in THP-1 cells with 100 nM FLNa siRNA or negative control siRNA as indicated. (**C**) Schematic model of CCL2-activated CCR2B in the cell membrane and during internalization. **1**) Dimerized FLNa interacts with the C-terminus of the CCR2B through at least repeat 19 and with β-arrestin through repeat 22. β-arrestin also binds the third intracellular loop of CCR2B. In addition, FLNa interacts with actin to crosslink actin fibers**. 2**) Vesicle-budding step of CCR2B-endocytosis. During CCL2-induced clathrin-mediated endocytosis of CCR2B, FLNa links the receptor and arrestin molecules to actin. Actin polymerization is triggered when the clathrin coat and the plasma membrane start to invaginate, and stops when vesicle scission occurs. **3**) Endogenous vesicles can be propelled through the cytoskeleton by actin comet tails, which might facilitate movement towards cytoskeletal structures or aid the fusion of endocytic organelles.

To investigate the requirement of FLNa in CCL2-induced THP-1 migration, we employed siRNAs to reduce the expression of endogenous filamin A. THP-1 cells were transfected with a non-specific control RNA and FLNa siRNA. Only cells transfected with FLNa RNAi showed a decrease in the levels of endogenous FLNa protein (Figure 9B). FLNa RNAi-transfected cells exhibited a 40%±9.15 reduction (n = 4) in the migration response to CCL2 compared to cells incubated with control siRNA (Figure 9A). These results indicate that selective inhibition of FLNa is sufficient to induce an impairment in CCL2-mediated migration of THP-1 cells.

## Discussion

The results presented demonstrate the association of FLNa with the CCR2B receptor and supports the idea that FLNa is responsible for the localization of the receptor in different dynamic membrane structures. This includes the protrusion of lamellipodia in migrating cells where receptor and FLNa protein colocalize and the internalization step of receptor endocytosis. The lack of FLNa not only disrupts receptor internalization but also the migration of monocytes on a chemokine gradient.

There are currently over 60 identified interaction partners for filamin A. These partners are of great functional diversity and range from membrane receptors and ion channels to transcription factors and intracellular signalling intermediates [11]. Of particular note is the fact that an increasing number of GPCRs have been shown to associate with FLNa, including D_2_ and D_3_ Dopamine receptors [17], [18], calcium sensing receptor [19], [20], metabotropic glutamate receptor type 7 [21], calcitonin receptor [22] and µ opioid receptor [23]. Unpublished results in our group suggest that the other splice variant of the CCR2 receptor, namely CCR2A, though it does not interact with the 19–24 domain of filamin A may interact with other regions of the proteins. The C-terminal tail region of FLNa is implicated in the majority of these interactions. We have demonstrated that the association of FLNa with the C-terminal tail of the CCR2B receptor comprises a region including the tandem repeat 19. A recent study has identified subgroups of filamin repeats (filamin subgroup A containing repeats 4, 9, 12, 17, 19, 21 and 23) that share a conserved ligand-binding site [24]. They also suggest a repeat/ligand interface. Curiously, CCR2B binds to at least one of the repeats of this group (repeat 19) and shares some sequence homology with the membrane receptors utilized in the analysis. Further mutation analysis of its sequence will help to elucidate the amino acids required for this interaction.

The interaction between filamin A and its protein partners plays various roles: regulation of surface expression, stabilization, clustering, degradation, internalization, recycling, anchoring to actin, sequestration and cell motility. So in principle, the interaction of the receptor with FLNa could fulfill several functions. Driving the receptor to the plasma membrane and/or stabilizing it would seem a likely possibility, but our results have been quite conclusive showing the presence of receptor in the plasma membrane of M2 melanoma FLNa-deficient cells and in HEK293 cells depleted of FLNa. On the contrary, the interaction between the stimulated receptor and FLNa is necessary for its internalization in endocytic vesicles and consequently it is not necessary for downstream calcium signaling. Cells depleted of FLNa show a blockage in ligand-induced internalization, while FLNa-deficient M2 cells display a reduced rate of internalization. During recent years, there has been increasing evidence in mammalian cells that the actin cytoskeleton participates directly in membrane dynamics during clathrin-mediated endocytosis [25]. Actin appears in clathrin-coated structures at the plasma membrane towards the end of the lifetime of the clathrin-coated pits, thereby overlapping with the process of vesicle internalization [26]. Actin polymerization is therefore tightly coupled, both spatially and temporarily, to the vesicle-budding step of endocytosis. On the other hand, it has been shown that invaginations form without actin recruitment in mammalian cells, but that efficient vesicle scission requires the involvement of actin [26]. In fact, our results agree with this model of internalization since the activated CCR2B receptor requires FLNa interaction to initiate internalization and probably the link to actin fibers. Besides binding to actin, FLNa will simultaneously interact with various proteins, others receptors or factors necessary for endocytosis. It is likely that by tethering the receptor to actin filaments, FLNa catalyzes the formation of CCL2–inducible complexes with interacting proteins that are necessary for the efficient endocytosis of the receptor into clathrin-coated pits (Figure 9C). Consequently, FLNa can function as a signaling platform, coordinating the association between multiple receptor/s and proteins localized at the budding vesicles and the actin structures.

One of the interesting recent findings has been the fact that β-arrestin proteins interact with FLNa [27], [28]. The β-arrestin binding site involves tandem repeat 22, which is close to the region of binding to other GPCRs, including CCR2B. The binding of FLNa to β-arrestin cooperates to activate the MAPK extracellular signal-regulated kinase (ERK) downstream of GPCRs. In the case of the D_3_R, the receptor is constitutively bound to both β-arrestin-2 and FLNa [27]. Interestingly, the authors have demonstrated that GRK activity acts as a rheostat to control the formation of D_3_R-β-arrestin-filamin complexes. Though we have no data proving the simultaneous interaction with the CCR2B, β-arrestin-2 and FLNa proteins, we have shown that the CCL2-stimulated receptor traffics in vesicles together with FLNa and also with β-arrestin-2. It is possible that FLNa may facilitate the interactions of the receptor with β-arrestin proteins (Figure 9C). In its absence, β-arrestin-2 still binds to the receptor, but there is a delay in the formation of clathrin coated pits and the internalization of the receptor, which could be due to the lack of simultaneous FLNa binding to both CCR2B and β-arrestin-2. In this way, FLNa could act as a major scaffolding protein for endocytic protein complexes.

As mentioned before FLNA interacts with several other GPCRs and membrane proteins. Though different roles have been given for the different receptor-FLNa interactions, it is interesting that FLNa affects the intracellular trafficking of various receptors. In the case of the µ opioid receptor, FLNa is implicated in its recycling and degradation [23]. Endocytic sorting and recycling of internalized calcitonin receptors are deficient in absence of FLNa [22]. This is also the case for the calcium sensing receptor [29]. FLNa has also been shown to affect transferrin receptor late endosome or lysosome targeting [30]. Though FLNa is crucial in the initial step of CCL2-induced vesicle formation, we have also observed the presence of activated receptor and FLNa in trafficking endosomes, and the incorrect localization of late endosomes in cells lacking FLNa which suggests that it may also be involved in other steps of CCR2B-vesicle trafficking besides the initial budding step of endosomes.

As mentioned before, the most likely mechanism by which FLNa affects the initial step of vesicle budding may involve the recruitment of other proteins together with the receptor to the endocytic machinery. We have attempted to analyze the implication of FLNa in receptor grouping at the membrane utilizing a cluster analysis, but we have not observed any difference in receptor distribution and cluster formation between FLNa-deficient and FLNa-repleted cells following ligand stimulation. For other receptors, FLNa is necessary for receptor clustering in the plasma membrane as is the case of dopamine D_2_ and D_3_ receptors [12]. For the D_3_R, FLNa is also essential for efficient sequestration from the membrane in a GRK2-dependent mechanism [27], [31]. FLNa also functions as a structural adaptor for CD4 and chemokine co-receptor clustering in T cells [32]. In the case of CCR2, another protein, FROUNT, has recently been shown to bind to the receptor at the membrane and the authors demonstrated that FROUNT is needed for receptor clustering [10]. So it may be possible that FROUNT is the major player in the receptor aggregation and that FLNa could act as a scaffold protein that connects the receptor to other downstream proteins, thereby facilitating both the formation and internalization of the clathrin coated pits as well as the activation of downstream signaling pathways.

The main function of chemokines is to induce the migration of cells to the sites of injury in the body. At the leading edge of the cell, the actin cytoskeleton is reorganized to form lamellipodia, generating a driving force for polarized migration. FLNa organizes cortical actin filaments and dynamic three-dimensional networks in the leading edges of migrating cells [10]. In fact, FLNa can regulate RhoGTPase-mediated cytoskeleton remodelling, which is for instance essential for monocyte migration. Here we find that CCR2B receptor concentrates with FLNa particularly in the leading edges of the membrane, more precisely, at the tip of actin filaments in lamellipodia structures. More importantly, our results demonstrate the requirement of FLNa for CCL2-induced cell migration. A quite intriguing observation is the presence of particles containing both receptor and FLNa along actin filaments spread at the leading edge. It should be noted that, substantial evidence is emerging suggesting the need of polarized trafficking during cell motility [33]. Thus, our data strongly suggest that FLNA could be responsible for localizing the receptor close to the actin remodeling proteins. In summary, FLNa may, in cooperation with other proteins, act as a scaffold for the spatial organization of ligand-activated chemokine receptors at the membrane at the leading edges of the cells by anchoring the receptor to actin filaments.

In conclusion, FLNa emerges as an important protein which links the CCR2B chemokine receptor to the actin filament structures and controls the fate of the stimulated receptors in dynamic membrane structures.

## Methods

### Cell culture

Human melanoma cell line M2 (lacking expression of FLNa) and isogenic cell line A7 (stably expressing full-length FLNa) (provided by J. Hartwig, Harvard Medical School, Boston, MA, USA) were maintained in α-minimal essential medium supplemented with 8% (v/v) newborn calf serum (NCS), 2% (v/v) fetal calf serum (FBS). A7 cells were cultured in the presence of 500 µg/ml G418. HEK293 cells were grown in Dulbecco's modified Eagle's medium (DMEM) supplemented with 10% (v/v) FBS and 250 µg/ml G418. Flag-CCR2B expressing HEK293 stable cells were made in the laboratory (S. Butt, University of Bergen). THP-1 cells (ATCC, Manassas, VA, USA) were maintained in RPM1 1640 medium supplemented with 0.05 mM 2-mercaptoethanol, and FBS (10%).

### DNA Constructs

A DNA sequence encoding a triple repetition of the terminal 47 residues of CCR2B (aa. 314–360) was cloned into the yeast expression plasmid pGBKT7 to generate pGBKT7-CCR2B47. The plasmid was constructed through a multistep procedure, essentially as described for the rhodopsin receptor [34]. The pMAL-cRI plasmid used was kindly provided by J–Z. Chuang and C–H. Sung (Cornell University Medical College, New York, USA). The pACT2-FLNar16–19 and pACT2-FLNar18 were cordially supplied by A. V. Binda (IBIOS, Pennsylvania State University College of Medicine, Hershey, PA, USA). Expression plasmids for repeats 23 and 24 of FLNa in pGADT7, pGADT7-FLNar23 and pGADT7-FLNar24 respectively, were made by PCR amplification using pREP4-FLNa (Dr J. Hartwig, Harvard Medical School, Boston, MA, USA) as a template. The pREP4-FLNa was also used for exogenous FLNa expression. The pcDNA3-FLAG plasmid was constructed by ligating the FLAG linker into pcDNA3 (Invitrogen, CA, USA), while pcDNA3-FLAG-CCR2B was made using the pcDNA3-ProFLAG-CCR2B [9].

### Yeast two-hybrid assay

The Matchmaker GAL4 Two-hybrid System 3 (Clontech, Saint-Germain-en-Laye, France) was used for the yeast two-hybrid studies. The pGBKT7-CCR2B47 plasmid was utilized to screen a human leukocyte Matchmaker cDNA library pre-transformed in *S. cerevisiae* strain AH109 under medium stringency selection conditions (synthetic dropout (SD) medium lacking Trp, Leu and His, supplemented with 2.5 mM 3- amino-1,2,4-triazole (3-AT)) following the manufacturer's instructions. Positive clones were multiple re-streaked on medium and high stringency selection media (SD medium lacking Trp, Leu, His and Ade, supplemented with 2.5 mM 3-AT and 4 mg/ml X-α-gal) before purification and sequencing of the harboured plasmids. Colonies sequentially co-transformed with pGBKT7-CCR2B47 and various constructs encoding FLNa repeats were plated out onto low stringency SD plates (lacking Trp and Leu). Single isolated colonies from each plate were picked, rigorously vortexed in 500 µl low stringency medium, from which 5 µl drops were placed onto low (SD/−2), medium (SD/−3) or high (SD/−4) stringency selection plates and incubated 3 days at 30°C.

### Cell transfections and immunoprecipitations

For transient transfections, Fugene HD (Roche Diagnostics, Basel, Switzerland) was used for the A7 and M2 cells, whilst Effectene (Qiagen, Hilden, Germany) was used for the HEK293 cells; both methods were implemented according to the manufacturers' instructions. For immunoprecipitations, cells were washed in ice-cold phosphate buffered saline (PBS) and solubilized in 1% Brij 96 V lysis buffer (20 mM Tris-HCl pH 7.5, 150 mM NaCl, 0.5 mM EGTA, 1 mM MgCl_2_), containing 1 mM Na_3_VO_4_, 50 mM NaF, 10 mM Na_4_P_2_O_7_ and a cocktail of protease inhibitors [35]. After clarification of the lysates, equal protein amounts were immunoprecipitated at 4°C overnight with M2 mouse anti-FLAG (Sigma-Aldrich Co., St. Louis, MO, USA), pre-coupled to Protein G-Sepharose beads. The immunocomplexes washed in 1% and 0.05% Brij lysis buffer. The immunoprecipitates and lysate controls were resolved on SDS-PAGE and transferred to nitrocellulose membranes. The presence of FLNa, CCR2B and β-actin was detected using anti-filamin 1 (Santa Cruz Biotechnologies, CA, USA), anti-FLAG (Sigma-Aldrich Co.) and anti-β-actin (Abcam, Cambridge, UK) antibodies, respectively. Blots were developed using an enhanced chemiluminescent method (Pierce, Thermo Scientific, Rockford, USA) and scanned using the LAS-3000 imaging system (Fujifilm, Tokyo, Japan).

### *In vitro* binding studies

Myc-CCR2B47 was expressed from pGBKT7-CCR2B47 using a TNT T7 Quick Coupled Transcription/Translation system (Promega Biotech AB, Nacka, Sweden) according to the manufacturer's instructions. Parental expression vector pGBKT7 was used as control. The *in vitro* reaction mixtures were mixed 1∶1 in 1% Brij 96 V lysis buffer. For binding studies, equal amounts of A7 or M2 cell lysates from untransfected cells were incubated with the *in vitro* translated pGBKT7 or pGBKT7-CCR2B47 mixture. Immunoprecipitation was performed by adding IgG-free bovine serum albumin (BSA) (Sigma-Aldrich Co.) and mouse anti-c-Myc (Zymed, CA, USA).

### Immunostaining and confocal microscopy

A7 and M2 cells were seeded on coverslips the day before. Immunofluorescence staining was done following different protocols: a) for whole cell staining, cells were washed in PBS, fixed with ice-cold 3% paraformaldehyde solution and permeabilized in PBS with 0.1% Tween 20 before staining with primary and secondary antibodies; b) to detect the receptor in the plasma membrane, cells were placed first on ice, incubated with primary antibodies before being fixed, permeabilized and incubated with secondary fluorescent antibodies; c) for internalization assays, cells treated first with anti-FLAG M2 antibodies (Sigma-Aldrich Co.) at 4°C were stimulated with 20 nM CCL2 (human MCP-1, PeproTech, Rocky Hill, NJ) at 37°C for the times indicated before being fixed, and incubated with secondary antibodies; d) to follow receptor remaining in the plasma membrane, cells were first CCL2-activated for the times indicated and fixed prior to treatment with anti-FLAG-Cy3 (Sigma-Aldrich Co.). After permeabilization, cells were incubated with anti-FLAG-FITC (Sigma-Aldrich Co.) antibodies. Other antibodies and dyes used were: rabbit anti-CCR2 (Abcam), Alexa Fluor 488 donkey anti-rabbit (Invitrogen), mouse anti-filamin 1, Texas Red goat anti-mouse (Invitrogen), FITC-phalloidin (Invitrogen) and Alexa Fluor 633 goat anti-mouse (Invitrogen). For the cluster analysis, complete z-stacks of the cells were taken and processed as maximum intensity projection. CellProfiler software [36] was used in order to determine and quantify the CCR2B particles in the cell surface. Data were processed by GraphPad Prism software and results were plotted representing the means ± SEM. Two-way ANOVA, Bonferroni or Mann Whitney tests were applied between cells and times (all the groups), times (0” vs. 30” vs. 60”) or stimulated cells (A7 vs. M2 without time 0”), respectively. To restore the A7 cell-like phenotype, M2 cells were transiently transfected with pcDNA3-FLAG CCR2B and pREP4-FLNa. The mean results of a total of 450 cells per treatment were plotted including ± SEM and a t-test was calculated with a p<0,05. Slides were mounted using Prolong Gold mounting medium with or without DAPI (Invitrogen). Optical sections were acquired using a Leica TCS SP2 AOBS confocal system equipped with a 63x Ibd.BL oil objective. Colocalization analyses were performed using the Imaris colocalization module (Bitplane AG, Zurich, Switzerland).

### Whole cell binding assay

Binding was carried out with (^125^I- Human MCP-1 (CCL2)), which was radio-iodinated using a standard protocol [37]. One day after transfection, cells were trypsinized and plated onto 12 well plates at a density of 5×10^4^ cells per well. The following day, cells were rinsed with HEPES-DMEM at 4°C, after which 0.5 ml of media containing 50 000 cpm of ^125^I- Human MCP-1 alone or together with increasing concentrations (10^−11^ M and 10^−5^ M) of unlabelled peptide were added to the cells. The cells were incubated at 4°C for 5 h, and subsequently washed with ice-cold PBS to remove unbound peptide. The cells were then solubilized with 0.5 ml 1 M NaOH for 10 min, transferred to counting tubes and counted in a γ-counter. The data was analyzed using GraphPad Prism software.

### CCR2B internalization assay in presence of FLNa siRNA

A synthetic FLNa-targeted siRNA oligoribonucleotide duplex with symmetric 3′TT overhang (FLNa siRNA sense sequence: 5′-AGGUGCUGCCUACUCAUGA-3′, [38]) was purchased from Dharmacon RNA Technologies (USA, Lafayette, CO). The following two oligoribonucleotides were used as negative control: siGENOME negative control siRNA #3 (Dharmacon), AllStars negative control siRNA (Qiagen). HEK293 cells stably expressing ProlactinFLAG-CCR2B were transiently transfected with 70 nM or 100 nM synthetic control siRNA or FLNa siRNA using Lipofectamine™ 2000 reagent (Invitrogen), and incubated 5 days at 37°C [38]. Cells were then washed in ice-cold PBS before being solubilized in 0.5% NP-40 lysis buffer (50 mM Tris, pH 7.4, 150 mM NaCl, and 0.5% NP-40) containing 1 mM EDTA, 0.1 mM Na_3_VO_4_ and a cocktail of protease inhibitors. After clarification, the lysates were resolved on SDS-PAGE and transferred to nitrocellulose membranes. Cells to be used for the internalization assay were trypsinised 4 days after transfection and replated onto poly-L-lysine-coated glass coverslips. After 24 h, cells were incubated with mouse anti-FLAG-M2-Cy3 antibody (Sigma-Aldrich Co.) at 4°C and stimulated with 20 nM CCL2 at 37°C for the times indicated before being fixed, and incubated with goat anti-FLNa (Santa Cruz Biotechnology, Inc.) followed by FITC-conjugated mouse anti-goat (Jackson ImmunoResearch, Suffolk, UK). 150 cells from each siRNA treatment per triplicate were quantified and classified for main presence of CCR2B receptor in the membrane or inside the cell. The mean results of a total of 450 cells per treatment were plotted in a chart indicating ± SEM and statistical t-test was calculated with a p<0,01.

### Chemotaxis assay in presence of FLNa siRNA

THP-1 cells were transiently transfected with 100 nM FLNa siRNA or siGENOME negative control siRNA #3 using the Nucleofector kit V or L (Amaxa) according to the manufacturer's instructions and incubated 5 days at 37°C. 100 000 cells from each sample were transferred to the upper chambers of a 24-well chemotaxis chamber (6.5 mm inserts, pore size 3 or 5 µm) (Corning, NY, USA) and 25 nM CCL2 in RPMI 1640 supplemented with 0.1% BSA was added to the lower chamber. After 24 h at 37°C, filters were removed and trespassed cells were quantified with the automatic detection mode in the Imaris software by analysing pictures of the wells taken under a NikonTE2000 microscope. Some of the samples treated with siRNA were lysed in 0.5% NP-40 lysis buffer and subjected to immunoblotting using rabbit anti-FLNa (Abcam) and anti-β-actin antibodies.

## Supporting Information

Figure S1 CCR2B cluster analysis in A7 and M2 cell surface. Three different cluster sizes (R1 = 0.07−0.14 µm; R2 = 0.14−0.5 µm and R3 = 0.5−3 µm) were analyzed for each time point and cell type. The graph represents the means ± SEM, n = 35 cells per cell type and p<0.05.
(0.24 MB TIF)

Figure S2 Colocalization of CCR2B with FLNa during CCL2 treatment. THP-1 (A) and CCR2B-A7 cells (B) were grown onto coverslips, incubated with rabbit anti-CCR2B or anti-FLAG-Cy3 and treated with 20 nM CCL2 for the times indicated. Cells were fixed, permeabilized and treated with anti-rabbit Alexa Fluor 488 or goat anti-mouse Alexa Fluor 568. Images are from one single layer of the Z stacks. The colocalization was analyzed using Imaris colocalization software and is shown in white. White dotted lines show the boundaries of the cells. Experiments were done in duplicates and repeated three times. Bars, 1 µm.
(0.92 MB TIF)

Figure S3 Filamin A is not necessary for downstream calcium signaling from CCL2-activated CCR2B. HEK293 cells stably expressing FLAG-CCR2B were left untreated or transiently transfected with 100 nM synthetic control siRNA or FLNa siRNA using Lipofectamine™ 2000 reagent for 5 days. Cells were then harvested and incubated for 20 min with 5 µM fluorescent calcium indicator Fluo-4 AM (Invitrogen) in DMEM with 10% FCS and 10 mM HEPES pH 7.4. Cells were washed, resuspended in PBS containing 2 mM CaCl2 and placed on ice. FACS acquisition was done using the Accuri C6 Flow cytometer and CFlowPlus software (Accuri Cytometers, Inc. Ann Arbor, MI USA). Samples were analyzed for 1 min to take the baseline, then for 5 min with 20 nM CCL2 and subsequently for 5 min with 2 µM ionomycin (transfected cells). Untransfected cells were left untreated (negative control) or treated with 2 µM ionomycin (Sigma-Aldrich Co.) (positive control). Data were analyzed using FLOWJO software version 7.6.0 (Tree Star, Inc. Ashland, OR USA) and are presented as Fluo-4 AM intensity over time. Experiments were done in duplicates and repeated twice.
(16.53 MB TIF)

Figure S4 β-arrestin-2 colocalizes with the CCL2 activated CCR2B in M2 and A7 cells. To follow CCR2B internalization and β-arrrestin-2 co-distribution, A7 and M2 cells double-transfected with pcDNA3-FLAG-CCR2B and β-arrestin-2-GFP were placed on ice, treated with anti-FLAG-Cy3 antibodies and stimulated with 20 nM CCL2. Images are representative of the majority of the cells and are from one single layer of the Z stacks. The colocalization was analyzed using Imaris colocalization software and is shown in white. White dotted lines show the boundaries of the cells. The experiment was repeated three times with similar results. Bars, 10 µm.
(1.14 MB TIF)
