# Supplementary figures and images for: Filamin A Binds to CCR2B and Regulates Its Internalization
Source: PLoS One. 2010 Aug 17;5(8):e12212. doi: 10.1371/journal.pone.0012212 (PMC2923182; doi:10.1371/journal.pone.0012212)

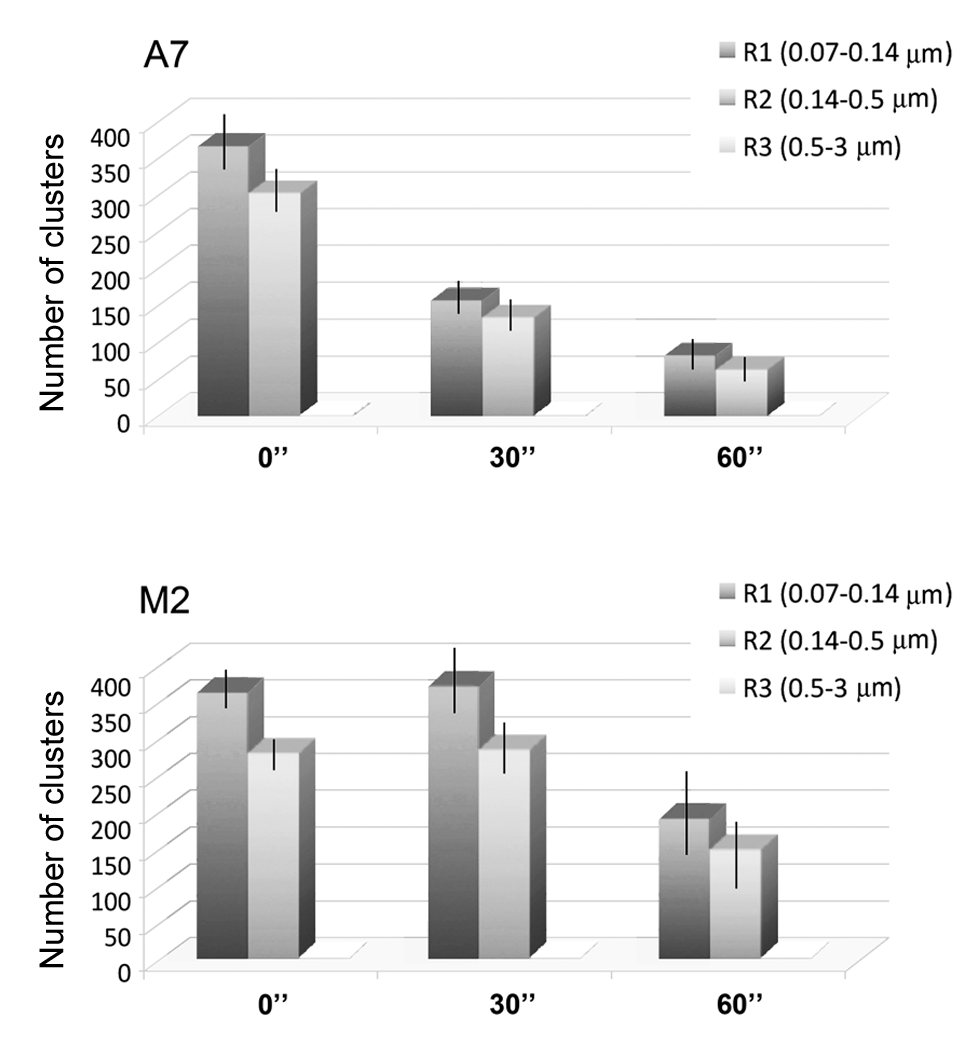

Supplement: Figure S1 — CCR2B cluster analysis in A7 and M2 cell surface. Three different cluster sizes (R1 = 0.07−0.14 µm; R2 = 0.14−0.5 µm and R3 = 0.5−3 µm) were analyzed for each time point and cell type. The graph represents the means ± SEM, n = 35 cells per cell type and p<0.05. (0.24 MB TIF) [file pone.0012212.s001.tif]

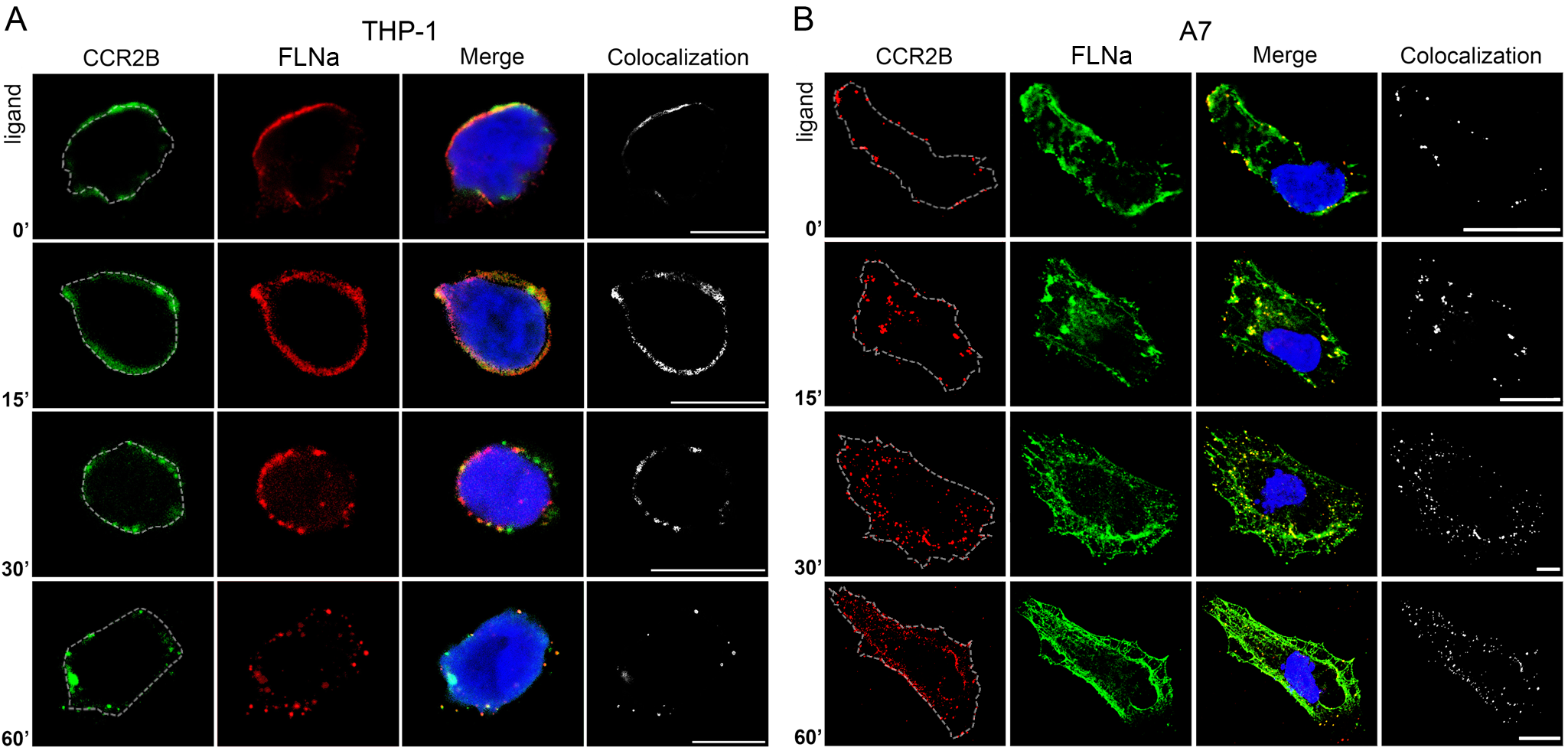

Supplement: Figure S2 — Colocalization of CCR2B with FLNa during CCL2 treatment. THP-1 (A) and CCR2B-A7 cells (B) were grown onto coverslips, incubated with rabbit anti-CCR2B or anti-FLAG-Cy3 and treated with 20 nM CCL2 for the times indicated. Cells were fixed, permeabilized and treated with anti-rabbit Alexa Fluor 488 or goat anti-mouse Alexa Fluor 568. Images are from one single layer of the Z stacks. The colocalization was analyzed using Imaris colocalization software and is shown in white. White dotted lines show the boundaries of the cells. Experiments were done in duplicates and repeated three times. Bars, 1 µm. (0.92 MB TIF) [file pone.0012212.s002.tif]

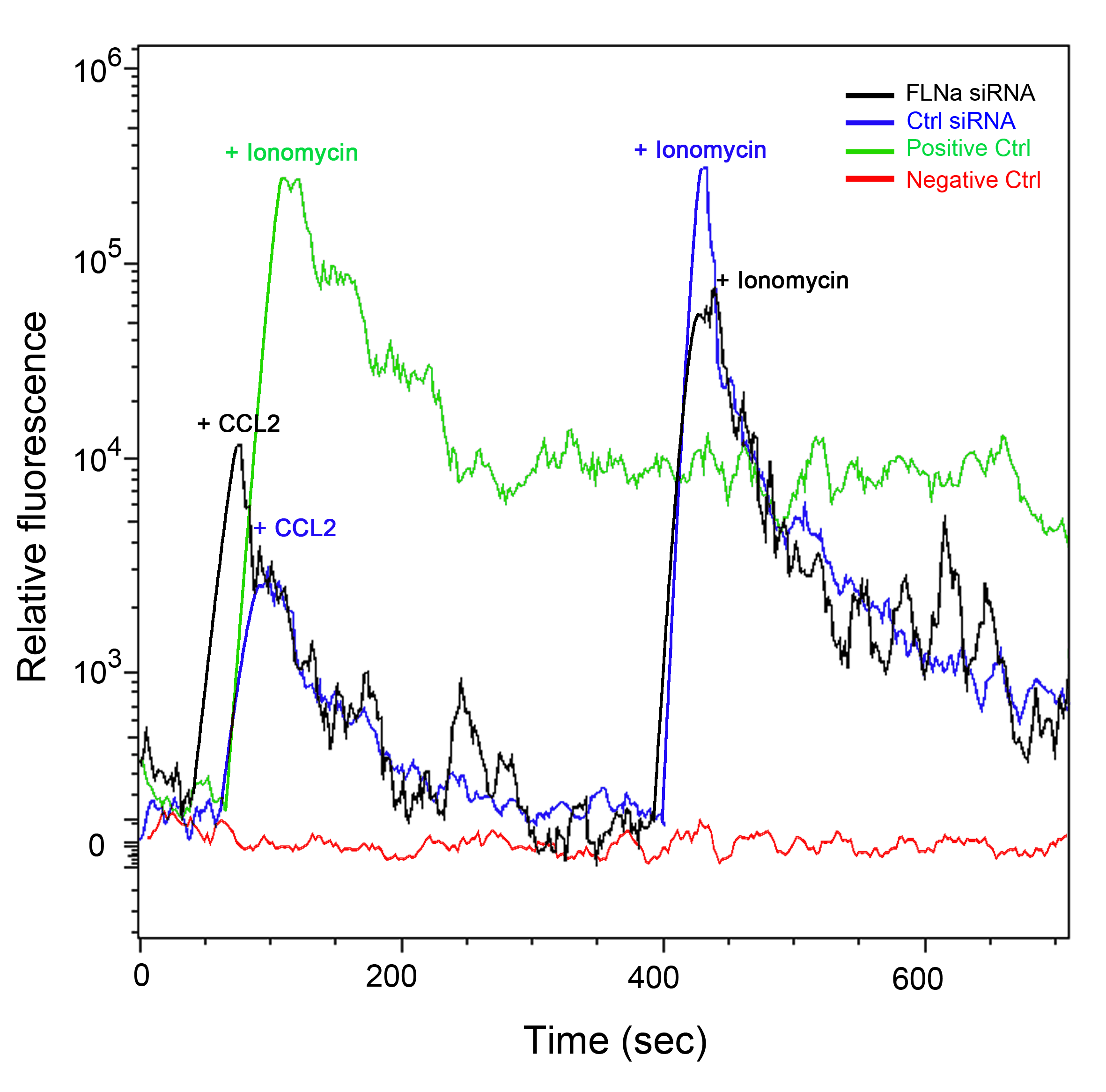

Supplement: Figure S3 — Filamin A is not necessary for downstream calcium signaling from CCL2-activated CCR2B. HEK293 cells stably expressing FLAG-CCR2B were left untreated or transiently transfected with 100 nM synthetic control siRNA or FLNa siRNA using Lipofectamine™ 2000 reagent for 5 days. Cells were then harvested and incubated for 20 min with 5 µM fluorescent calcium indicator Fluo-4 AM (Invitrogen) in DMEM with 10% FCS and 10 mM HEPES pH 7.4. Cells were washed, resuspended in PBS containing 2 mM CaCl2 and placed on ice. FACS acquisition was done using the Accuri C6 Flow cytometer and CFlowPlus software (Accuri Cytometers, Inc. Ann Arbor, MI USA). Samples were analyzed for 1 min to take the baseline, then for 5 min with 20 nM CCL2 and subsequently for 5 min with 2 µM ionomycin (transfected cells). Untransfected cells were left untreated (negative control) or treated with 2 µM ionomycin (Sigma-Aldrich Co.) (positive control). Data were analyzed using FLOWJO software version 7.6.0 (Tree Star, Inc. Ashland, OR USA) and are presented as Fluo-4 AM intensity over time. Experiments were done in duplicates and repeated twice. (16.53 MB TIF) [file pone.0012212.s003.tif]

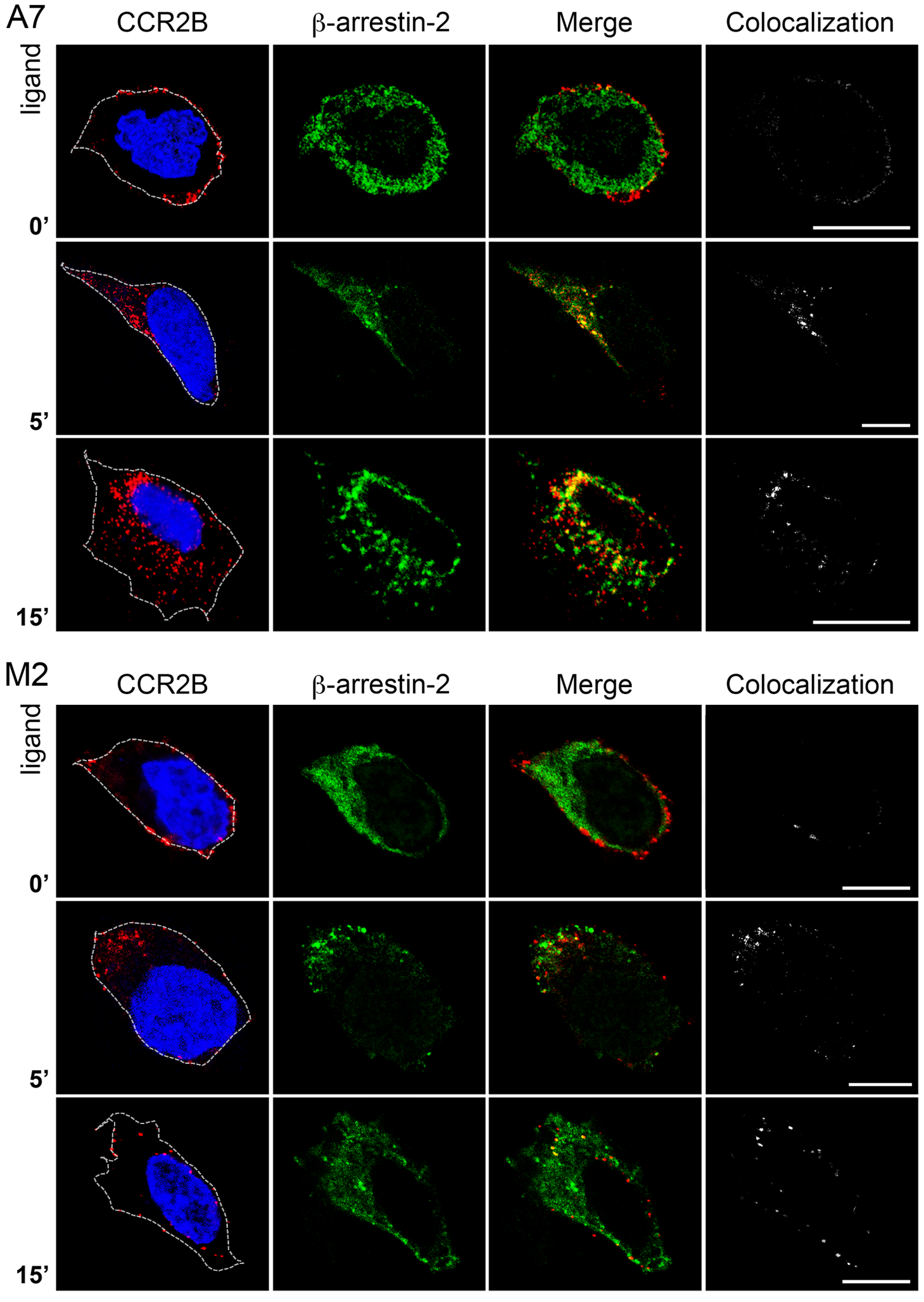

Supplement: Figure S4 — β-arrestin-2 colocalizes with the CCL2 activated CCR2B in M2 and A7 cells. To follow CCR2B internalization and β-arrrestin-2 co-distribution, A7 and M2 cells double-transfected with pcDNA3-FLAG-CCR2B and β-arrestin-2-GFP were placed on ice, treated with anti-FLAG-Cy3 antibodies and stimulated with 20 nM CCL2. Images are representative of the majority of the cells and are from one single layer of the Z stacks. The colocalization was analyzed using Imaris colocalization software and is shown in white. White dotted lines show the boundaries of the cells. The experiment was repeated three times with similar results. Bars, 10 µm. (1.14 MB TIF) [file pone.0012212.s004.tif]
